# Modified Smoothing Algorithm for Tracking Multiple Maneuvering Targets in Clutter

**DOI:** 10.3390/s22134759

**Published:** 2022-06-23

**Authors:** Sufyan Ali Memon, Min-Seuk Park, Imran Memon, Wan-Gu Kim, Sajid Khan, Yifang Shi

**Affiliations:** 1 Department of Defense Systems Engineering, Sejong University, Seoul 05006, Korea; ms.park@sejong.ac.kr (M.-S.P.); kimwangu@sejong.ac.kr (W.-G.K.); 2 Department of Computer Science, Bahria University, Karachi Campus, Karachi 74200, Pakistan; imranmemon.bukc@bahria.edu.pk; 3 Department of Computer Science, IBA Sukkur University, Sukkur 65111, Pakistan; sajidkhan@iba-suk.edu.pk; 4 School of Automation, Hangzhou Dianzi University, Hangzhou 310018, China; syf2008@hdu.edu.cn

**Keywords:** component existence probabilities, false-track discrimination, multi-maneuvering-targets, smoothing, target existence probabilities

## Abstract

This research work extends the fixed interval smoothing based on the joint integrated track splitting (FIsJITS) filter in the multi-maneuvering-targets (MMT) tracking environment. We contribute to tackling unknown dynamics of the multi-maneuvering-targets (MMT) using the standard kinematic model. This work is referred to as smoothing MMT using the JITS (MMT-sJITS). The existing FIsJITS algorithm is computationally more complex to solve for the MMT situation because it enumerates a substantial number of measurement-to-track assignments and calculates their posteriori probabilities globally. The MMT-sJITS updates a current target track by assuming the joint (common) measurements detected by neighbor tracks are modified clutters (or pretended spurious measurements). Thus, target measurement concealed by a joint measurement is optimally estimated based on measurement density of the modified clutter. This reduces computational complexity and provides improved tracking performance. The MMT-sJITS generates forward tracks and backward tracks using the measurements collected by a sensor such as a radar. The forward and backward multi-tracks state predictions are fused to obtain priori smoothing multi-track state prediction, as well as their component existence probabilities. This calculates the smoothing estimate required to compute the forward JITS state estimate, which reinforces the MMT tracking efficiently. Monte Carlo simulation is used to verify best false-track discrimination (FTD) analysis in comparison with existing multi-targets tracking algorithms.

## 1. Introduction

We investigate the multi-maneuvering-target (MMT) tracking problem which attained huge awareness in recent studies. Many MMT tracking systems use infinite resolution sensors such as radar and sonar to track targets in a difficult, cluttered environment [1]. However, these types of sensor do not have prior knowledge on the target which has a low probability of detection $P_{D}$. The sensor detects and produces uncertain measurements returned by both the real target and the other random object’s sources (such as reflections from terrain). The clutter measurements returned by the random sources concealed the targets so that the surveillance scenario becomes complex.

The multi-target tracking (MTT) algorithms generate the tracks subjected to the available sensor measurements with consequences in the true track (real target) and the false track (clutter). To discriminate the tracks, a widely used false-track discrimination (FTD) technique was developed which mainly identified the detection of targets to confirm the target tracks [2]. The FTD calculates a track quality measure based on the target existence probability. In [3], the authors have developed the algorithm referred as an integrated probabilistic data association (IPDA) which standardized the equation of the target existence probability. The conventional multi-target algorithms such as the joint probabilistic data association [4,5], joint IPDA (JIPDA) [6], and the joint integrated track splitting (JITS) [7] develop a cluster of multi-tracks and detected joint measurements. The joint data (measurement) association implies the shared (common) measurements associated to different tracks. The joint measurement does not guaranteed that a measurement belongs to a target; in fact, if it finds and detects a target measurement, there are always possibilities of changing the measurement to another target. Therefore, this joint data association method enumerates a substantial number of measurements-to-tracks assignments and obtains the corresponding probabilities of detected measurements as well as their data associations. The number of assigned hypotheses extend in a combinatorial way cardinality with a number of cluster tracks and their measurements. This makes the MMT system practically difficult to implement. In addition, the JITS employed an integrated track splitting filter (ITS) [8] in cluster formation and evaluation. The ITS filter partitions a track into number of components which grows exponentially in each scan. Another multiple hypothesis tracking method was developed in [9], which finds the global measurement-to-associations by considering all measurements and tracks, and thus develops a large number of measurement hypothesis. These intractable mathematical complexities were solved using the linear multi-target (LM) tracking approach [2]. The LM is a suboptimal MTT algorithm which directly converts single target tracking method such as IPDA and ITS into the MTT algorithm by incorporating the joint measurements as a modified clutter measurements. In brief, the target detection measurement being followed by neighbor tracks acts as modified clutter and thus, a current track state estimation is updated in the coordinates of modified clutter measurement. For example, the LM based on the IPDA and ITS were investigated in [10,11,12]. Except [4,5,9], all reference algorithms provide the measure of track quality for FTD evaluation.

We apply the smoothing method to improve the target state estimate in the past scan by using the measurements received from upcoming scans compromising a predefined smoothing-time delay [2,13]. The most widely used smoothing approaches subjected to the standard joint probabilistic data association algorithm [14], multiple hypothesis smoothing filter [15], and smoothing with probability density [16] were invented in the multi-target tracking situation but they lack the capability to achieve FTD. Many researchers developed smoothing algorithms using the JIPDA and JITS without discussing the maneuvering dynamics of the targets such as the fixed-interval smoothing subjected to the JIPDA (FIsJIPDA) [17] and to the JITS (FIsJITS) [18]. Both FIsJIPDA and FIsJITS outperformed the earlier fixed-interval smoothing JIPDA [19] and smoothing JITS [20].

We have investigated that the optimal smoothing methods in the MTT environment, such as FIsJIPDA and FIsJITS, face a lot of mathematical complexities due to the procedure of joint data association. Therefore, in this paper, we integrate the FIsJITS feasible joint measurement events (FJEs) with the LM method by considering joint events as modified clutter measurements and develop a new fixed measurement interval smoothing algorithm called smoothing multi-maneuvering-targets based on JITS (MMT-sJITS). This new contribution makes the proposed MMT-sJITS simple in a algorithmic structure with less computational complexity. The MMT-sJITS employ JITS in forward-time direction (fJITS) and backward-time direction (bJITS) separately to fuse their predictions. The MMT-sJITS generates tentative forward JITS (fJITS) tracks and develops a validation gate based on the backward measurements without using the sensor measurements, because the backward measurements are predicted subject to the sensor measurements. In contrast with existing algorithms, the MMT-sJITS does not use joint data association for MMT state estimation. The priori smoothing predictions followed by fusion process are obtained to select the smoothing validation measurements from the sensor set of measurements. These smoothing measurements often consist of the joint measurements scenario; therefore, the weighted posteriori smoothing probabilities of these joint smoothing measurements are evaluated to determine modified clutter measurement densities in the coordinates of the current detected measurement. This calculates both the MMT-sJITS and fJITS track estimates in each scan. Ultimately, both the MMT-sJITS and fJITS track are refined using the smoothing validation measurements. The MMT-sJITS track becomes robust due to fJITS track reinforcement in each scan for MMT situation in clutter. For algorithm implementation, we employ the standard nearly constant velocity kinematic model without utilizing any computationally expensive type of maneuvering models (such as discussed by [21]). Monte Carlo simulations are used to verify the smoothing and FTD results of MMT-sJITS in cluttered environment.

## 2. Target Trajectory Model

We have assumed that the target state $x_{k}^{\tau }$ (where τ denotes the label of the target as well as the track and *k* denotes the scan index) is a random variable and the target existence $\chi_{k}^{\tau }$ is a random probabilistic event. It is also assumed that a tracking sensor such as radar receives at most one measurement per radar scan with a low probability of target detection $P_{D}$. Without loss of generality, we supposed that a MMT tracking system measures position and velocity vectors in the two-dimensional surveillance environment. However, there is no limitation to utilize the algorithm in the three-dimensional environment, such as the one which was developed based on the fixed lag smoothing IPDA in [22]. The MMT system estimates the target state $x_{k}^{\tau }$ without a priori information on its maneuvering dynamics. The τth target state prediction linearly propagates in each scan *k*; for example, the state prediction propagating from k−1 to *k* is expressed by:

(1) $$x_{k}^{\tau }=F_{k-1}x_{k-1}^{\tau }+v_{k-1},$$

where $F_{k-1}=T\left[I_{2\times 2},I_{2\times 2};O_{2\times 2},I_{2\times 2}\right]$ denotes the state propagation matrix, $v_{k-1}$ denotes target model white noise that has a zero mean and a covariance $Q_{k-1}$, *T* denotes a scan time, $I_{2\times 2}$ indicates the 2×2 identity matrix, and $O_{2\times 2}$ indicates the 2×2 zeros matrix. The sensor measures a target position in each scan *k* by:

(2) $$z_{k}^{\tau }=H_{k}x_{k}^{\tau }+w_{k},$$

where $H_{k}=\left[I_{2\times 2},O_{2\times 2}\right]$ denotes state position measurement matrix and $w_{k}$ represents white Gaussian sensor noise that has a zero mean and a covariance $R_{k}$.

The measurement set collected by a sensor in kth scan is represented by $Y_{k}$. Due to measurement uncertainties in the cluttered environment, the sensor could lose a target and follow a clutter. The clutter measurement is formed using a non-homogenous Poisson method [23] that contaminates the target measurement. We have assumed a known clutter measurement density of the ith measurement $Y_{k,i}$; that is, $\rho_{k,i}\equiv \rho \left(Y_{k,i}\right)$.

## 3. Smoothing Joint Integrated Track Splitting Algorithm

**Figure 1:**
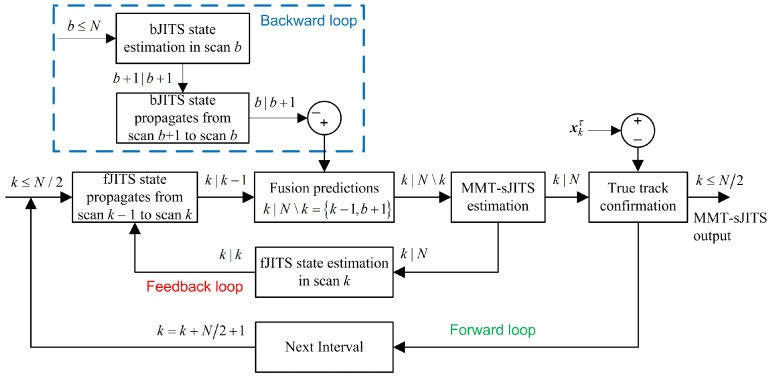
Block diagram of the MMT-sJITS algorithm.

This section proposes a new integrated smoothing method which integrates the FIsJITS using a modified clutter measurement density to smooth the multi-maneuvering-targets in clutter (referred as MMT-sJITS algorithm). An important contribution of the proposed method is to improve the weakness of the FIsJITS algorithm for MMT in clutter. We have assumed the overlapped measurement interval [17], which consists of N−k+1 scans. The block diagram of the MMT-sJITS resembles a feedback tracking loop which obtains the smoothing state estimate to estimate a forward track state estimate, as illustrated in Figure 1. The MMT-sJITS uses the following subscript notations, which define the scan index and conditioning on the available measurements:

- b≤N: *b* denotes the backward scan index and *N* denotes the last scan index of the fixed interval [b:N];
- b+1|b+1 denotes the current scan and current scan measurement in a backward direction; for example, ${\hat{x}}_{b+1|b+1}^{\tau ,c}$ (where, superscript τ,c denotes the track component and an accentˆ(hat) indicates a state estimate) is the backward state estimate obtained in scan b+1 conditioning on the measurements set $Y_{b+1}$;
- b|b+1 denotes the next scan time *b*, but conditioned on the previous backward scan b+1; for example, ${\bar{x}}_{b|b+1}^{\tau ,c}$ (where, an accent¯(bar) indicates a state prediction) is the backward state prediction obtained based on the measurements set $Y_{b+1}$ and propagated to the next scan *b*;
- k|k−1 denotes the next scan *k* in forward direction but conditioned on the previous scan k−1; for example, ${\bar{x}}_{k|k-1}^{\tau ,c}$ is the forward state prediction obtained in previous scan k−1 based on the previous scan measurements set $Y_{k-1}$, which now propagated to the next scan *k*;
- k|N\k denotes a current forward scan and conditioning on the last scan measurements set $Y_{N}$ up to $Y_{k+1}$, and not including measurements set of scan $Y_{k}$, which is used to show the fusion of forward and backward multi-tracks (e.g., ${\bar{x}}_{k|N\backslash k}^{\tau ,c}$ is the fused priori smoothing state prediction in scan *k*, conditioned on $Y_{N}$ excluding $Y_{k}$);
- k|N denotes the current scan in a forward direction, conditional on the last scan measurements set $Y_{N}$ up to current scan measurements set $Y_{k}$; for example, ${\hat{x}}_{k|N}^{\tau ,c}$ is the smoother state estimate obtained in scan *k* conditioning on the measurements set up to $Y_{N}$, including the measurements set $Y_{k}$;
- k|k denotes the current scan and conditioning on current scan measurements set $Y_{k}$ in a forward direction; for example, ${\hat{x}}_{k|k}^{\tau ,c}$ is the forward state estimate obtained in scan *k* conditioning on the measurements set $Y_{k}$;
- k≤N/2 indicates that the smoothing state statistics is evaluated for up to half of the fixed interval [k:N/2,N/2+1:N], so that the next overlapped fixed interval can be processed starting from scan k=k+N/2+1.

Figure 1 clearly illustrates the flow of the MMT-sJITS algorithm. First, the backward loop is generated to obtain the bJITS multi-track state estimation and prediction recursively from the Nth scan to bth scan. Later, fJITS initializes each one of the forward multi-tracks with corresponding state prediction in scan k−1 and propagates it to the fusion block. In the fusion, each fJITS state prediction develops a validation gate in the coordinates of the validated backward state prediction, assuming all backward multi-track state predictions as a set of measurements under the fJITS framework in scan k|N\k. This produces the multiple numbers of true pairs of fusion components, referred to as priori smoothing component predictions, which are associated with the validated backward measurements. These priori smoothing predictions select a subset of the smoothing validation measurements from the measurements set $Y_{k}$ to obtain the MMT-sJITS state estimation as well as fJITS state estimation. A feedback loop is generated to update the fJITS based on the smoothing statistics, which produces the forward multi-track state prediction at scan k−1. Similarly, a feedback-loop continues until scan k≤N. At each scan *k* in the interval [k:N/2], a chi-squared statistical test is performed to verify the true value of the smoothed state estimate [2]. Finally, following the loop of next overlapped measurement interval k+N/2+1, we obtained the MMT-sJITS output statistics recursively in each scan *k*.

### 3.1. Backward Joint Integrated Track Splitting (bJITS)

The MMT-sJITS applies the JITS [7] algorithm in the reverse chronological order of the measurement interval [b,N], where *b* indicates a backward scan. An interval consists of $Y^{b}=\left[Y_{b},Y_{b+1},\ldots ,Y_{N}\right]$ measurements set, where $Y_{N}$ denotes the measurements set collected by a sensor in the last scan index *N* of the corresponding interval. The backward tracks are initialized using each pair of measurements collected from two successive sets of measurements (two-point distance method [2]). The tracks are propagated from scan b+1 to scan *b* in the form of track components using the following Kalman filter propagation equation [24,25]:

(3) $$\begin{array}{c} {\bar{x}}_{b|b+1}^{\tau ,c}=F_{b+1}^{-1}{\hat{x}}_{b+1|b+1}^{\tau ,c}\\ {\bar{P}}_{b|b+1}^{\tau ,c}=F_{b+1}^{-1}{\hat{P}}_{b+1|b+1}^{\tau ,c}F_{b+1}^{-t}+Q_{b+1} \end{array},$$

where $F_{b+1}=F_{k-1}^{-1}$, $Q_{b+1}=F_{k-1}^{-1}Q_{k-1}F_{k-1}^{-t}$, and superscript *t* indicates a transpose. Each new backward track carries an initial probability of target existence $P\{\chi_{b+1}^{\tau }|Y_{b+1}\}$ and an initial component existence $\zeta_{b+1|b+1}^{\tau ,c}=1$.

The probability of target existence is a track quality measure with respect to a track τ, which is recursively updated and propagated using a Markov Chain One model [2,3], that is defined by:

(4) $$P\left\{\chi_{b}^{\tau }|Y_{b+1}\right\}=\alpha P\left\{\chi_{b+1}^{\tau }|Y_{b+1}\right\},$$

where α represents the state transition probability of the target which implies the time update of the priori probability of target existence event from scan b+1 to the current scan *b*.

Each bJITS creates a validation gate in the bth scan using measurement selection criteria [2] so that each τth track collects a subset of the components validation measurements $y_{b,i}^{\tau ,c}$ from the set of measurement $Y_{b}$ using:

(5) $${\left(Y_{b,i}-H_{b}{\bar{x}}_{b|b+1}^{\tau ,c}\right)}^{t}{\left(S_{b}\right)}^{-1}\left(Y_{b,i}-H_{b}{\bar{x}}_{b|b+1}^{\tau ,c}\right)\leq \gamma ,$$

where $S_{b}=H_{b}{\bar{P}}_{b|b+1}^{\tau ,c}H_{b}^{t}+R_{b}$ denotes the covariance of the sensor measurement residual and γ denotes the maximum threshold of the validation gate which is determined by the gating probability $P_{G}={\left(1-e\right)}^{-0.5\gamma }$ [2,13]. The value of $P_{G}$ should be maximum such as 0.999, which corresponds to the threshold value of 13.5. Thus, a residue of the measurements set in the coordinates of the state prediction must be equal to or below the threshold 13.5 to select the subset of validation component measurements from the set of measurements received from the sensor. Because the algorithm does not know priori information on the sensor measurements, therefore, the threshold 13.5 is kept the same for both target as well as clutter tracks. The selected component validation measurement computes the corresponding component likelihood as follows:

(6) $$\begin{array}{c} l_{b,i}^{\tau ,c}=\frac{1}{\sqrt{2\pi |S_{b}}|}e^{-0.5{\left(y_{b,i}^{\tau ,c}-H_{b}{\bar{x}}_{b|b+1}^{\tau ,c}\right)}^{t}S_{b}^{-1}\left(y_{b,i}^{\tau ,c}-H_{b}{\bar{x}}_{b|b+1}^{\tau ,c}\right)}. \end{array}$$

Equation (6) is used to calculate the backward track likelihood of the selected measurement $y_{b,i}^{\tau ,c}$ as expressed by:

(7) $$l_{b,i}^{\tau }=\sum_{c^{\tau }}\zeta_{b|b+1}^{\tau ,c}l_{b,i}^{\tau ,c}.$$

The bJITS creates a cluster of tracks where the measurements are mapped to the tracks using Equation (8) and form a feasible joint event (FJE) $\epsilon_{i}$ for i≥0. The bJITS allocates only joint (common) measurements within one cluster and uncommon measurements to another cluster, so that each cluster is processed independently [19]. Therefore, MMT-sJITS ignores the entire joint data association evaluation method of the FIsJITS and obtains the weighted posterior probability of the FJE with respect to the τth track using the Equation (8). Here, for i=0, $l_{b,i}^{\tau }=0$, and thus, $p\left(\epsilon_{i}^{\tau }|Y_{b}\right)=G^{-1}\prod_{\tau \in t_{0}\left(\epsilon_{i}^{\tau }\right)}\left(1-P_{D}P_{G}P\left\{\chi_{b}^{\tau }|Y_{b+1}\right\}\right)$.

(8) $$\begin{array}{c} p\left(\epsilon_{i}^{\tau }|Y_{b}\right)=G^{-1}\prod_{\tau \in t_{0}\left(\epsilon_{i}^{\tau }\right)}\left(1-P_{D}P_{G}P\left\{\chi_{b}^{\tau }|Y_{b+1}\right\}\right)\times \\ \;\;\;\;\;\;\;\;\;\;\;\;\;\;\;\;\;\;\prod_{\tau \in t_{i}\left(\epsilon_{i}^{\tau }\right)}\left(P_{D}P_{G}P\left\{\chi_{b}^{\tau }|Y_{b+1}\right\}\frac{l_{b,i}^{\tau }/\rho_{b,i}}{\sum_{i=1}^{m_{b}}l_{b,i}^{\tau }/\rho_{b,i}}\right) \end{array},$$

where $t_{0}$ indicates that the measurement was not selected by a track and $t_{1}^{i}$ indicates that the track has a ith measurement, $m_{b}$ indicates the number of validation measurements selected by Equation (5) and *G* represents the normalized constant factor that must satisfies:

(9) $$\sum_{\epsilon_{i}^{\tau }}\left\{\epsilon_{i}^{\tau }|Y_{b}\right\}=1.$$

The modification in the probabilistic equation (obtained in Equation (8)) avoids the influence of the measurements originated from other targets by assuming them as modified (pretended) clutter measurements. This significantly reduces the computational complexity in MMT-sJITS algorithm. Consider that the cluster track labeled with σ (τ∉σ) is acting as modified clutter track in a cluster with a modified clutter measurement density expressed by:

(10) μb,iτ=ρb,i+∑σ=1σ≠τσ=τnlb,iσpϵiσ|Yb1−pϵiσ|Yb,

which is calculated in the coordinates of the validated measurement $y_{b,i}^{\tau ,c}$ with respect to the τth track, $\tau_{n}$ represents the number of cluster tracks. Equation (10) is used to compute the backward track likelihood ratio $\lambda_{b}^{\tau }$ for i>0 as expressed in Equation (11). However, for i=0, $\lambda_{b}^{\tau }=1-P_{D}P_{G}$.

(11) $$\lambda_{b}^{\tau }=1-P_{D}P_{G}+P_{D}P_{G}\sum_{i>0}\frac{l_{b,i}^{\tau }}{\mu_{b,i}^{\tau }}.$$

Each ith measurement is a mutually exclusive, so that only one validated measurement corresponds to the potential τth target with a posteriori probability of target existence given by:

(12) $$P\left\{\chi_{b}^{\tau }|Y_{b}\right\}=\frac{\lambda_{b}^{\tau }P\left\{\chi_{b}^{\tau }|Y_{b+1}\right\}}{1-\left(1-\lambda_{b}^{\tau }\right)P\left\{\chi_{b}^{\tau }|Y_{b+1}\right\}}.$$

Simultaneously, each validated measurement forms a new track component with corresponding component existence probability expressed by:

(13) $$\zeta_{b|b}^{\tau ,c}=\frac{\zeta_{b|b+1}^{\tau ,c}}{\Lambda_{b}^{\tau }}\left\{\begin{array}{cc} \;1-P_{D}P_{G}; & if\;i=0\\ \;P_{D}P_{G}\frac{l_{b,i}^{\tau ,c}}{\mu_{b,i}^{\tau }}; & if\;i>0 \end{array}\right.,$$

The predicted bJITS track components (obtained from Equation (3)) are estimated using the Kalman filter equation of estimation [24,25]:

(14) $$\begin{array}{c} {\hat{x}}_{b|b,i}^{\tau ,c}={\bar{x}}_{b|b+1}^{\tau ,c}+K_{b}\left(y_{b,i}^{\tau ,c}-H_{b}{\bar{x}}_{b|b+1}^{\tau ,c}\right)\\ {\hat{P}}_{b|b,i}^{\tau ,c}={\bar{P}}_{b|b+1}^{\tau ,c}-K_{b}H_{b}{\bar{P}}_{b|b+1}^{\tau ,c} \end{array},$$

where $K_{b}={\bar{P}}_{b|b+1}^{\tau ,c}H_{b}^{t}S_{b}^{-1}$ represents the Kalman gain measured in scan *b*. Similarly, the backward multi-tracks state estimations are calculated recursively using Equations (3)–(14) in each scan *b*.

### 3.2. Forward Joint Integrated Track Splitting (fJITS)

When backward scan *b* arrives at the first scan *k* scan of an interval [b≥k,N], the MMT-sJITS initializes the multi-tracks using a forward-running JITS (fJITS). The fJITS uses a two-point distance [2] method to initialize the tracks based on two successive measurement sets; that is, $Y^{k}=\left[Y_{k-1},Y_{k}\right]$. Each fJITS track carries an initial probability of target existence $P\{\chi_{k-1}|Y_{k-1}\}$ and an initial probability of component existence $\zeta_{k-1|k-1}^{\tau ,c}=1$. The fJITS track components state propagates to scan *k* using Equation (15).

(15) $$\begin{array}{c} {\bar{x}}_{k|k-1}^{\tau ,c}=F_{k-1}{\hat{x}}_{k-1|k-1}^{\tau ,c}\\ {\bar{P}}_{k|k-1}^{\tau ,c}=F_{k-1}{\hat{P}}_{k-1|k-1}^{\tau ,c}F_{k-1}^{t}+Q_{k-1} \end{array}.$$

The fJITS creates a validation gate by exploiting backward multi-track components state predictions calculated in scan k=b as the measurements set. Thus, the measurement selection criteria [2] are used to validate a backward component for fusion in a forward track. That is:

(16) $${\left({\bar{x}}_{b|b+1}^{\tau ,c}-{\bar{x}}_{k|k-1}^{\tau ,c}\right)}^{T}{\left(s_{k}\right)}^{-1}\left({\bar{x}}_{b|b+1}^{\tau ,c}-{\bar{x}}_{k|k-1}^{\tau ,c}\right)\leq \gamma ,$$

where $s_{k}=\left({\bar{P}}_{b|b+1}^{\tau ,c}-{\bar{P}}_{k|k-1}^{\tau ,c}\right)$ denotes the covariance of the backward measurement residual. As a result, the MMT-sJITS produces a selected number of validated pairs composed of forward and backward component state predictions for fusion in the validation gate. The priori smoothing track component state prediction accompanied by its covariance associated to the validated backward track τ are expressed by the following equations [26]:

(17a) $$\begin{array}{cc} {\left({\bar{P}}_{k|N\backslash k}^{\tau ,c}\right)}^{-1} & ={\left({\bar{P}}_{b|b+1}^{\tau ,c}\right)}^{-1}+{\left({\bar{P}}_{k|k-1}^{\tau ,c}\right)}^{-1} \end{array},$$

(17b) $$\begin{array}{cc} {\bar{x}}_{k|N\backslash k}^{\tau ,c} & ={\bar{P}}_{k|N\backslash k}^{\tau ,c}\left[{\left({\bar{P}}_{k|k-1}^{\tau ,c}\right)}^{-1}{\bar{x}}_{k|k-1}^{\tau ,c}+{\left({\bar{P}}_{b|b+1}^{\tau ,c}\right)}^{-1}{\bar{x}}_{b|b+1}^{\tau ,c}\right] \end{array}.$$

In addition, if any of the bJITS track fails to satisfy Equation (16), then it becomes equal to an associated forward track state component prediction. That is, $\left[x_{k|N\backslash k}^{\tau ,c},P_{k|N\backslash k}^{\tau ,c}\right]=\left[x_{k|k-1}^{\tau ,c},P_{k|k-1}^{\tau ,c}\right]$. Each fused state component calculates the smoothing priori component existence probability by utilizing the associated priori bJITS track state component probability in the following equation [26]:

(18) $$\zeta_{k|N\backslash k}^{\tau ,c}=\frac{\zeta_{k|k-1}^{\tau ,c}}{\Lambda_{N\backslash k}^{\tau }}\left\{\begin{array}{c} \;1-P_{D}^{\tau }P_{G};if\;\tau \;is\;not\;validated\\ \;P_{D}^{\tau }P_{G}\zeta_{b|b+1}^{\tau ,c}\frac{l_{N\backslash k,i}^{\tau ,c}}{f_{b,i}^{\tau }};if\;\tau \;is\;validated \end{array}\right..$$

where $f_{b,i}^{\tau }=\tau_{n}/A$ is the assumed total number of backward tracks per unit area of the surveillance region A, and $P_{D}^{\tau }=1-{\left(1-P_{D}^{\tau }\right)}^{N-k+1}$ represents the assumed probability of target existence that determines the existence probability either in k+1 or in k−1. Based on these assumptions, the priori smoothing track likelihood ratio $\lambda_{N\backslash k}^{\tau }$ with respect to fusion track τ is expressed by:

(19) $$\lambda_{N\backslash k}^{\tau }=1-P_{D}P_{G}+P_{D}P_{G}\sum_{i>0}\frac{l_{N\backslash k,i}^{\tau }}{f_{b,i}^{\tau }},$$

where $l_{N\backslash k,i}^{\tau }$ represents the likelihood of the fusion track in the coordinates of the selected backward measurement. We have:

(20) $$l_{N\backslash k,i}^{\tau }=\sum_{c^{\tau }}\zeta_{k|N\backslash k}^{\tau ,c}l_{N\backslash k,i}^{\tau ,c},$$

where $l_{N\backslash k,i}^{\tau ,c}$ represents the hybrid component likelihood measurement of fJITS and bJITS paired components as expressed by:

(21) $$\begin{array}{c} l_{N\backslash k,i}^{\tau ,c}=\frac{1}{\sqrt{2\pi |s_{k}}|}e^{-0.5{\left({\bar{x}}_{b|b+1}^{\tau ,c}-{\bar{x}}_{k|k-1}^{\tau ,c}\right)}^{t}s_{k}^{-1}\left({\bar{x}}_{b|b+1}^{\tau ,c}-{\bar{x}}_{k|k-1}^{\tau ,c}\right)} \end{array}.$$

The MMT-sJITS calculates a priori smoothing τth target existence probability by using Equation (19) in:

(22) $$\begin{array}{c} P\left\{\chi_{k}^{\tau }|Y_{N\backslash k}\right\}=\frac{\Lambda_{N\backslash k}^{\tau }P\left\{\chi_{k}^{\tau }|Y_{k-1}\right\}P\left\{\chi_{b}^{\tau }|Y_{b+1}\right\}}{1-\left(1-\Lambda_{N\backslash k}^{\tau }\right)P\left\{\chi_{k}^{\tau }|Y_{k-1}\right\}P\left\{\chi_{b}^{\tau }|Y_{b+1}\right\}} \end{array}.$$

Equation (22) modifies the conventional equation of the probability of target existence [2,3] by utilizing the τth potential target existence probability that was calculated by a validated backward track. The reason behind it is obvious since the τth target was already identified in the bth scan using the bJITS iteration followed by Equation (14).

### 3.3. MMT-sJITS Smoothing Track Update

The MMT-sJITS uses Equation (17) in the validation selection criteria [2] to find the residual of the sensor measurements in the validation gate and selects a subset of smoothing track component validation measurements ${\tilde{y}}_{k,i}^{\tau ,c}$ (where tilde accent denotes smoothing) from the set of $Y_{k}$ by using:

(23) $${\left(Y_{k}-{\bar{x}}_{k|N\backslash k}^{\tau ,c}\right)}^{t}{\left({\tilde{S}}_{k}\right)}^{-1}\left(Y_{k}-{\bar{x}}_{k|N\backslash k}^{\tau ,c}\right)\leq \gamma ,$$

where ${\tilde{S}}_{k}=H_{k}{\bar{P}}_{k|N\backslash k}^{\tau ,c}H_{k}^{t}+R_{k}$ represents the covariance of the measurement residual. Each feasible smoothing validated component measurement ${\tilde{y}}_{k,i}^{\tau ,c}$ has a smoothing component likelihood ${\tilde{l}}_{k,i}^{\tau ,c}$ with respect to the track τ as expressed by:

(24) $$\begin{array}{c} {\tilde{l}}_{k,i}^{\tau ,c}=\frac{1}{\sqrt{2\pi |{\tilde{S}}_{k}}|}e^{-0.5{\left({\tilde{y}}_{k,i}^{\tau ,c}-H_{k}{\bar{x}}_{k|N\backslash k}^{\tau ,c}\right)}^{t}{\tilde{S}}_{k}^{-1}\left({\tilde{y}}_{k,i}^{\tau ,c}-H_{k}{\bar{x}}_{k|N\backslash k}^{\tau ,c}\right)} \end{array},$$

which computes the smoothing track likelihood ${\tilde{l}}_{k,i}^{\tau }$ of the smoothing measurement:

(25) $${\tilde{l}}_{k,i}^{\tau }=\sum_{c}\zeta_{N\backslash k}^{\tau ,c}{\tilde{l}}_{k,i}^{\tau ,c}.$$

The MMT-sJITS maps feasible outcomes ${\tilde{y}}_{k,i}^{\tau ,c}$ to the smoothing track by making the feasible joint measurement events and evaluates their weighted a posteriori probabilities. That is, if the τth target is detected and its measurement $z_{k}^{\tau }$ is selected in the validation gate ($z_{k}^{\tau }\in {\tilde{y}}_{k,i}^{\tau ,c}$) then the weighted smoothing a posteriori probabilities of the feasible joint measurement events $\tilde{p}\left(\epsilon_{i}^{\tau }|Y_{N}\right)$ being followed by a track τ is computed by using Equations (22) and (25) in Equation (8) replacing $P\left\{\chi_{b}^{\tau }|Y_{b+1}\right\}$ and $l_{b,i}^{\tau ,c}$, respectively. The MMT-sJITS uses $\tilde{p}\left(\epsilon_{i}^{\tau }|Y_{N}\right)$ and ${\tilde{l}}_{k,i}^{\tau }$ to obtain the smoothing modified clutter measurement density being observed by a smoothing track τ. We have:

(26) μ˜k,iτ=ρk,i+∑σ=1σ≠τσ=τnl˜k,iσp˜(ϵiσ|YN)1−p˜(ϵiσ|YN).

Similar to the bJITS iteration, the MMT-sJITS calculates a posteriori smoothing τth target existence probability by using:

(27) $$P\left\{\chi_{k}^{\tau }|Y_{N}\right\}=\frac{{\tilde{\lambda }}_{k}^{\tau }P\left\{\chi_{k}^{\tau }|Y_{N\backslash k}\right\}}{1-\left(1-{\tilde{\lambda }}_{k}^{\tau }\right)P\left\{\chi_{k}^{\tau }|Y_{N\backslash k}\right\}},$$

where ${\tilde{\lambda }}_{k}^{\tau }$ represents the likelihood ratio of the smoothing track which is obtained by using Equations (25) and (26) in Equation (11) replacing $l_{b,i}^{\tau }$ and $\mu_{k,i}^{\tau }$, respectively.

The MMT-sJITS uses Equation (17) in Equation (14) (replacing ${\bar{x}}_{b|b+1}^{\tau ,c}$ and ${\bar{P}}_{b|b+1}^{\tau ,c}$) based on the validated smoothing component measurements ${\tilde{y}}_{k,i}^{\tau ,c}$ (replacing $y_{k,i}^{\tau ,c}$ in Equation (14)) to obtain smoothing component state estimate $x_{k|N}^{\tau ,c}$ and its corresponding covariance $P_{k|N}^{\tau ,c}$. Finally, the posteriori smoothing probability of the component existence is calculated similar to Equation (13); that is:

(28) $${\tilde{\zeta }}_{k|N}^{\tau ,c}=P_{D}P_{G}\frac{\zeta_{N\backslash k}^{\tau ,c}{\tilde{l}}_{k,i}^{\tau ,c}}{{\tilde{\mu }}_{k,i}{\tilde{\lambda }}_{k}^{\tau }}.$$

However, if i=0, then ${\tilde{\zeta }}_{k|N}^{\tau ,c}=\zeta_{k|N\backslash k}^{\tau ,c}$. Equation (28) is utilized to approximate the MMT-sJITS track components using a Gaussian probability density function mean and its covariance, respectively. We have:

(29a) $${\hat{x}}_{k,N}^{\tau }=\sum_{c}{\tilde{\zeta }}_{k|N}^{\tau ,c}{\hat{x}}_{k|N,i}^{\tau ,c},$$

(29b) $${\hat{P}}_{k,N}^{\tau }=\sum_{c}{\tilde{\zeta }}_{k|N}^{\tau ,c}\left({\hat{P}}_{k|N,i}^{\tau ,c}+{\hat{x}}_{k|N,i}^{\tau ,c}{\left({\hat{x}}_{k|N,i}^{\tau ,c}\right)}^{t}\right)-{\hat{x}}_{k,N}^{\tau }{\left({\hat{x}}_{k,N}^{\tau }\right)}^{t},$$

where subscript k,N indicates that the MMT-sJITS track state estimate is computed in scan *k* based on the measurements provided up to scan *N* in an interval [k,N]. Equation (29) is obtained recursively in each scan *k* to compute the MMT-sJITS output track state statistics.

Since the clusters are already formed in forward path track with the MMT-sJITS update, the steps including measurement selection, FJE formation and posteriori probabilities of joint measurement events are not required for the forward track state estimation. The MMT-sJITS calculates the state estimate of the fJITS track components by utilizing the smoothing component measurements (selected from Equation (23)) and updates the fJITS track using the modified smoothing clutter measurement densities (obtained in Equation (26)). Hence, refining the fJITS tracks in a smoothing fashion; that is, they are attaining more accurate information regarding the τth target state and state existence. The fJITS computes the track state component likelihood $l_{k,i}^{\tau ,c}$ of the measurement ${\tilde{y}}_{k,i}^{\tau ,c}$ in the coordinates of the corresponding fJITS track state component prediction ${\bar{x}}_{k|k+1}^{\tau ,c}$ by applying Equation (24) with subscript k|k−1 on the component prediction. In addition, the likelihood of the fJITS track is a weighted multiplication of the smoothing component existence probabilities and the forward track state components likelihoods as expressed by:

(30) $$l_{k,i}^{\tau }=\sum_{c}\zeta_{k|N}^{\tau ,c}\sum_{c}\zeta_{k|k-1}^{\tau ,c}l_{k,i}^{\tau ,c}.$$

Equations (26) and (30) are applied in Equation (11) to obtain the likelihood ratio $\lambda_{k}^{\tau }$ of the fJITS track, which is used to obtain the posteriori τth target existence probability as expressed by:

(31) $$P\left\{\chi_{k}^{\tau }|Y_{k}\right\}=\frac{\lambda_{k}^{\tau }P\left\{\chi_{k}^{\tau }|Y_{k-1}\right\}}{1-\left(1-\lambda_{k}^{\tau }\right)P\left\{\chi_{k}^{\tau }|Y_{k-1}\right\}}.$$

The fJITS track components state estimation such as ${\hat{x}}_{k|k}^{\tau ,c}$ and its state covariance ${\hat{P}}_{k|k}^{\tau ,c}$ are obtained by using the smoothing component measurements ${\tilde{y}}_{k,i}^{\tau ,c}$ and the predicted fJITS state components (${\bar{x}}_{k|k+1}^{\tau ,c}$ and ${\bar{P}}_{k|k+1}^{\tau ,c}$ obtained from Equation (15)) in the Kalman Filter Equation (14). Consequently, these updated components are applied to Equation (15) for recursive fJITS track propagation in each scan *k*. Each updated fJITS track state component calculates its updated component existence probability by utilizing the Equations (26) and (28) in the following Equation:

(32) $$\zeta_{k|k}^{\tau ,c}=P_{D}P_{G}\frac{{\tilde{\zeta }}_{k|N}^{\tau ,c}l_{k,i}^{\tau ,c}}{{\tilde{\mu }}_{k,i}\lambda_{k}^{\tau }},$$

Thus, each fJITS track becomes a powerful tool for treating the bJITS multi-tracks as measurements and associating the backward predictions for fusion in the forward validation gate. This not only improves the fusion of forward and backward validated predictions but also enhance the capability of the smoothing algorithm for an efficient multi-maneuvering-target tracking in the subsequent scans.

### 3.4. Implementation of the MMT-sJITS Algorithm

**Figure 2:**
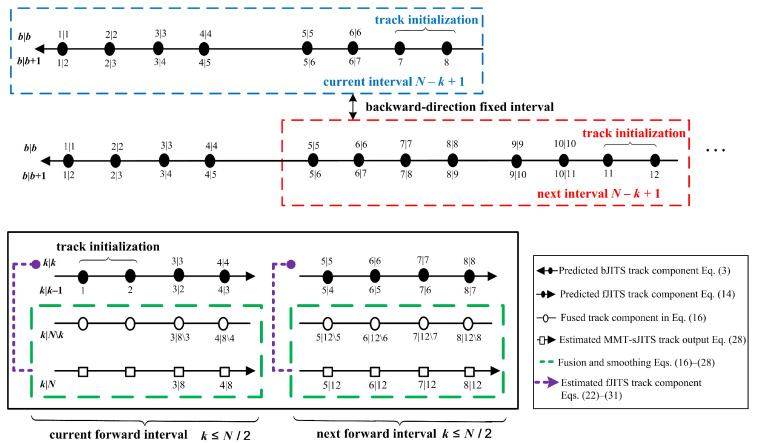
Fixed overlapped measurement interval (Est—estimation; Pred—prediction; Fus—fusion).

The proposed MMT-sJITS algorithm is implemented using the fixed overlapped measurement interval, which consists of N−k+1 scans [17,22] as illustrated in Figure 2.

Let the current fixed measurement interval starts from scan k=1 and end with scan k=8; that is, the total length of this fixed interval is supposed to be N−k+1=8 scans as depicted by a blue dotted-rectangular box in Figure 2. The larger the length of measurement interval, the better the estimation statistics. The following describes the three basic steps used in the implementation of the fixed smoothing interval structure:

1. bJITS procedure is illustrated in the blue rectangular frame showing the iteration from the first fixed interval. bJITS multi-track state component estimation and prediction are computed recursively in each scan starting from the Nth to bth scan, followed by Equations (3)–(14). The indices of backward scans and conditioning on the measurements (both old and new conditioned observation data) are described in Section 3 (see Figure 1). For example, ${\hat{x}}_{6|6}^{\tau ,c}$ is a state component estimate and ${\bar{x}}_{6|7}^{\tau ,c}$ is a state component prediction. Note that the last two scans (i.e., N−1 and *N*) are used to initialize backward tracks (two-point initialization approach [2]) in every interval.
2. fJITS multi-tracks are initialized using two successive scan measurements (e.g., k=1 and k=2) [2]. fJITS track state component prediction is computed using Equation (15), which propagates linearly and performed a fusion corresponding to the selected bJITS track components in each scan index k|N\k (e.g., ${\bar{x}}_{3|8\backslash 3}^{\tau ,c}$).
3. MMT-sJITS multi-track smoothing state estimate is obtained in scan *k* conditioning on $Y_{N}$, which indicates that the state is updated based on all measurements collected up to the Nth scan, including the current scan *k* (e.g., ${\hat{x}}_{3|8}^{\tau ,c}$). Consequently, the validated smoothing measurements are applied to the fJITS via feedback loop to calculate the forward multi-track state estimation in each scan using Equations (16)–(32). Here, ${\hat{x}}_{3|3}^{\tau ,c}$ is state estimation conditioning on $Y_{k}$ and on scan *k* which is propagated using Equation (15) to obtain the state component prediction in scan k=4 conditioning on $Y_{3}$, such as, ${\bar{x}}_{4|3}^{\tau ,c}$. ${\bar{x}}_{3|2}^{\tau ,c}$ is a state prediction obtained in scan k=3 conditioning on scan k=2 and on $Y_{2}$ using the track initialization process.

In each forward-time scan, the newly intialized fJITS are concatenated with existing fJITS tracks so that both the new and estimated fJITS track components are propagated simultaneously for fusion in each scan. The maximum smoothing is obtained when we overlapped the current fixed measurements interval to the next interval. For example, we have overlapped the interval at 5th scan as indicated by a red rectangular frame in Figure 2; that is, the first half of the interval is smoothed and stored to obtain the output statistics, and the next half is overlapped to the next four subsequent scans, making the same length of interval (12−5+1=8). In the next fixed interval, k=5,6,…,12, bJITS restarts the backward iteration using Equations (3)–(14) to obtain the multi-tracks state estimation from scan b=12 to b=5. Similarly in the next interval, MMT-sJITS obtains the smoothing and forward multi-tracks estimate in each scan and continues the overlap smoothing interval procedure as depicted in Figure 2. At the end of the simulation, the MMT-sJITS smooths all consecutive scans in the last measurement interval. The overlapping of the fixed interval not only limits the smoothing time-delay but also maximizes the smoothing performance.

## 4. MMT-sJITS Analysis Using Simulation

The false track discrimination (FTD), root-mean square error (RMSE), and the statistics of the multi-track retention of the proposed MMT-sJITS method are compared with the existing MTT algorithms such as FIsJITS, JITS, FIsJIPDA, and JIPDA.

### 4.1. Track Component Management

The MMT-sJITS method applies the track component merging and pruning [2,26,27,28] to manage the growing length of forward and backward components. These track management approaches are not part of the algorithm; therefore, further details on both approaches can be found in the references. The fJITS and bJITS compared the component measurement directories from the four latest scans and merged the identical component measurement using one Gaussian probability density function mean and its covariance. The MMT-sJITS imposed a predefined pruning limit to delete the track components with a low component existence probabilities that are determined from $\zeta_{k|k}^{\tau ,c}$ and $\zeta_{b|b}^{\tau ,c}$ in forward and backward track, respectively. Because, smoothing components does not propagate, therefore, component management procedure is not required in smoothing tracks. In the simulation analysis, we used the same component management approach in the MMT-sJITS, FIsJITS and JITS algorithms.

### 4.2. False Track Discrimination (FTD)

Without loss of generality, a tentative track becomes a confirmed track if its updated track existence probability excelled by the confirmation threshold; or else becomes a terminated track if its updated track existence probability sank by a track deletion threshold [29]. The MMT-sJITS utilizes the smoothed target existence probabilities to determine the track quality measure required for FTD evaluation. Similarly, both fJITS and bJITS applied the FTD method to determine the quality of their tracks. Only, confirmed fJITS and bJITS tracks are used in the evaluation of the MMT-sJITS multi-tracks statistics. Therefore, the updated target existence probabilities of bJITS, MMT-sJITS, and fJITS are calculated recursively by applying Equations (12), (27) and (31) in each scan. For a fair analysis of the algorithm, a confirmed track remains confirmed until its updated track existence probability dips below a deletion threshold. Because of the influence of the non-homogeneous extended clutters, a confirmed track often reflected by a clutter. Therefore, a chi-squared statistical criterion [2,13] is applied to the status of the confirmed track as expressed by:

(33) $$\begin{array}{c} {\left({\hat{x}}_{k,N}^{\tau }-x_{k}^{\tau }\right)}^{T}{\left(P_{0|0}^{\tau }\right)}^{-1}\left({\hat{x}}_{k,N}^{\tau }-x_{k}^{\tau }\right)<\gamma \end{array},$$

where $P_{0|0}^{\tau }$ is the initialized covariance determined from the sensor measurement noise and γ represents the selection threshold that depends on the false-alarm probability of chi-squared distribution [2,13]. The two consequences are as follows:

1. Confirmed true tracks (CTTs): To achieve a CTT, a confimed track must satisfy Equation (33) with γ≤20. The CTTs stay in the same status until their normalized distance squared value (expressed by parenthesis term in above Equation) goes ahead of the maximum threshold limit; that is, γ≥40.
2. Confirmed false track (CFTs): With γ>20, a confirmed track becomes a CFT. It is also possible CFT becomes a CTT and vice versa. The value of γ is not fixed and can be used differently, which depends on the situation of the surveillance scenarios and requirements.

We assumed a point target tracking; therefore, at most one measurement belongs to a target track. Therefore, we have utilized the auction algorithm [30] to identify and detect only one target measurement by a CTT in each scan. The auction reference method computes the weighted score of the normalized distance squared of the target state corresponding to each CTT. Each CTT passes a bid and computes the weighted score to determine the winning bid. Thus, a CTT is associated to the τth target state smoothed estimate with a highest bid. Further details on the use of auction method can be found from the reference.

### 4.3. Output Statistics Analysis

**Figure 3:**
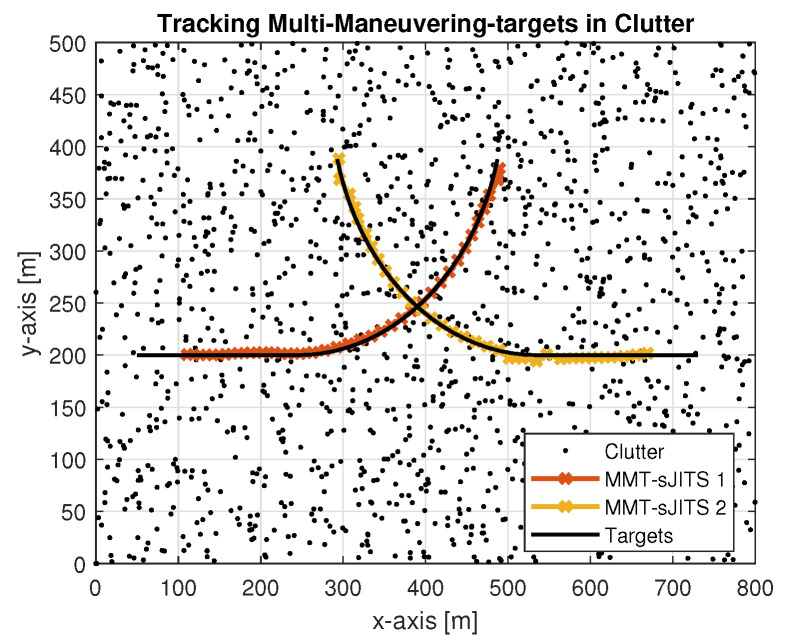
Trajectories of multi-maneuvering targets in cluttered environment.

We assume that two maneuvering targets are moving in the two-dimensional cluttered environment, which is corrupted with random number of clutters with the measurement density of $\rho_{k,i}=1\times 10^{-4}m^{-2}$, as shown in Figure 3.

The surveillance platform depicted in Figure 3 has a length and a width dimension of 800 m and 500 m along the *x*-axis and *y*-axis, respectively. A sensor produces a measurement noise covariance $R_{k}=25I_{2\times 2}$ and tries to detect the targets with $P_{D}=0.9$. There are 36 scans with a scan time of T = 1 s composed in 500 Monte Carlo runs. Around 29,205 (58 per run in average) false forward and 39,944 (80 per run in average) false backward tracks were generated using the two-point measurement distance equation [2] with the assumed maximum velocity of target $V_{\max}$ = 25 m/s. The two-point distance equation measured the uniform velocity of each target, which approximately equals 15 m/s until the scan k=14. Later, each target is reflected by the target maneuvering dynamic corresponding to the coordinated turn under the assumption:

(34) $$F_{M}=\left[\begin{array}{cccc} 1 & 0 & \sin\omega T/\omega & -1-\cos\omega T/\omega \\ 0 & 1 & 1-\cos\omega T/\omega & \sin\omega T/\omega \\ 0 & 0 & \cos\omega T & -\sin\omega T\\ 0 & 0 & \sin\omega T & \cos\omega T \end{array}\right],$$

where $F_{M}$ denotes the maneuvering state propagation and ω denotes the coordinated turn rate. Note that the algorithm does not know the value of the angular velocity of the target ω. The turning rate velocity was calculated based on the nearly constant velocity model, as expressed in Equations (3) and (15).

Each algorithm including the MMT-sJITS assumes the Markov Chain One model [2,3] of the target existence event, so that the tracks are recursively updated and propagated using the state propagation probability α=0.98, in each scan *k*. Furthermore, assume that a low initial probability of target existence 0.01 is assigned to track at its initialization scan and the confirmation threshold of the target existence is varied to obtain almost a similar number of confirmed false tracks (≈20) in each algorithms.

**Figure 4:**
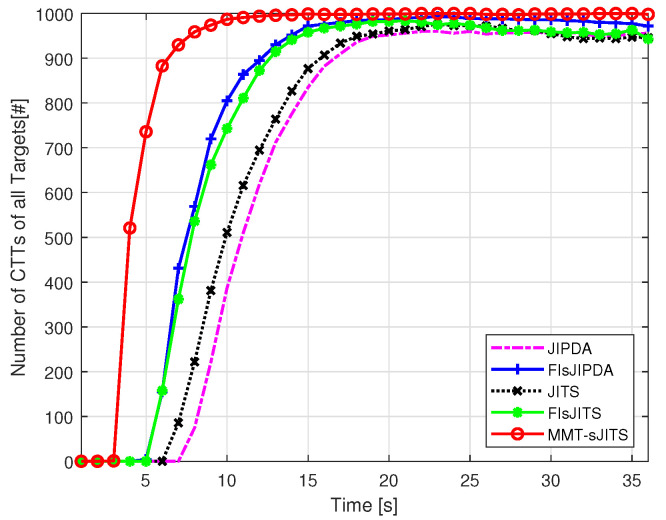
Total number of confirmed true tracks (CTTs).

**Figure 5:**
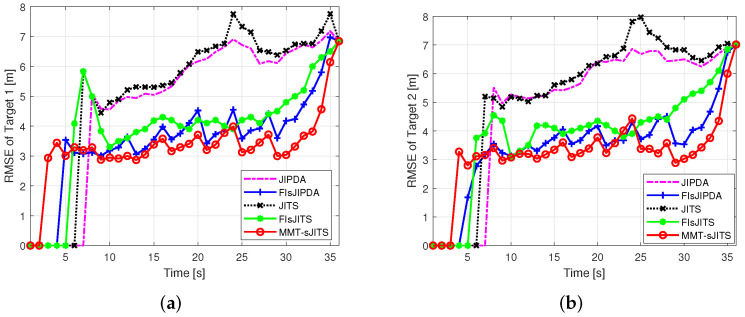
Root mean square errors (RMSEs) (**a**) RMSE of Target 1 (**b**) RMSE of Target 2.

Figure 4 shows tangible FTD comparison of the algorithms. The MMT-sJITS confirms the track (target) in scan k=4 and shows the highest number of the confirmed true tracks (CTTs). While other methods are lacking the improvement in the estimation which results in higher position root-mean square estimated errors (RMSEs) as depicted in Figure 5a,b. The FIsJIPDA, FIsJITS, and MMT-sJITS utilize the smoothing measurements for their forward and smoothing update, which results in good FTD performance compared to others. However, we can see the RMSEs statistics of the FIsJIPDA and FIsJITS are higher than that of MMT-sJITS as depicted in Figure 5. Thus, the MMT-sJITS provides almost 100% efficiency in tracking from scan 10 to end scan and shows a reduced estimation error as shown in Figure 4 and Figure 5, respectively. The conventional non-smoothing reference methods such as JIPDA and JITS result in the highest RMSEs. Finally, Figure 5 shows the convergence of the algorithms at end scan k=36 when their estimates taper off near the end scan since they have used same set of measurements in the end scan for their iterations.

An important conclusion from the simulation results presented in this paper and from the one which was illustrated in FIsJITS [17] is that the execution time of FIsJITS exponentially increases as the number of targets increases. For example, the execution time required for FIsJITS to track three targets and five targets is 3.0 s and 7.6 s, respectively, as discussed in [17]. In addition, the FIsJITS forms the clusters for all tracks and all measurements in [17]. In this paper, the FIsJITS allocates only joint measurements within one cluster and non-joint (non-shared) measurements to another cluster; each cluster was processed independently [19]. This mainly limits the computation time but degrades the performance of the tracking system due to unknown maneuvering dynamics of the targets. In the comparison, the proposed MMT-sJITS integrates the MMT system with LM method [10] and utilizes the modified clutter measurement (which plays the role of joint measurements) to update the current target-track. Thus, the MMT-sJITS acts like a single target tracker which significantly improved the tracking performance of the unknown targets in a cluttered environment.

The track retention statistics of all algorithms are determined by storing the identity number of the CTTs corresponding to each target in scan k=17. We need to show how much number of CTTs retained with the same track identity until scan k=30? This implies that the track identity of CTTs is checked before and after crossing of the multi-targets. Each track is identified by track index τ. The track retention statistics are accumulated from 500 Monte Carlo simulation runs, as shown in Table 1.

In the Table 1, we can define the following parameters:

- Case: a number of CTTs followed by a τth target in scan k=17;
- Okay: a number of CTTs pursued the same τth target in scan k=30;
- Swapped: a number of CTTs exchanged by some other target CTT τ in scan 30;
- Lost: a number of CTTs in scan 30 lost due to either track deletion because of the low target existence or low component existence probabilities, or they became CFTs;
- Result: a number of CTTs in the final scan k=36.
- Execution time [s]: the average calculation time per each Monte Carlo Simulation run that was analyzed using the MatLab R2020b software on the platform, 11th Intel Core™i7-1165G7 (@ 2.80 GHz, 2.80 GHz).

Table 1 shows the highest number of Case, Okay, and Result, and the lowest number of Lost in the MMT-sJITS algorithm. In the comparison, the JITS has the lowest number of Swapped, but it also has the lowest number of Case and Okay compared to the proposed method. The MMT-sJITS has consumed more time (1.64 s) per run compared to that of consumed by the JITS because of the smoothing-time delay. However, MMT-sJITS provides the significant performance in terms of multi-track retention and FTD. The FIsJITS and FIsJIPDA digest highest CPU time per run because they were involved in lengthy calculations of joint measurements data associations. The FIsJITS has some limitations due to the mathematical complexities in the algorithm. Therefore, imposing the FIsJITS to track the multi-targets in difficult surveillance situations may stop the simulation at a certain stage due to excess memory in the available platform source. The proposed MMT-sJITS method allowed the MMT system to avoid the enumeration of joint measurement assignments while assuming them as modified or pretended clutters. Therefore, the computational complexity in the MMT-sJITS is approximately linear in terms of the number of targets and the number of measurements involved. The reason behind the highest number of Case, Okay, and Result, and the lowest number of Lost in the MMT-sJITS, is due to its capability to avoid the track allocation of the joint measurement associations. In addition, the MMT-sJITS confirms the track at earliest and provides a rapid growth of smoothed target existence probabilities in each scan. Thus, a rapid increase in the number of CTTs was observed in the simulation results.

## 5. Conclusions

The MMT-sJITS extends the FIsJITS method in a multi-maneuvering-targets scenario. Compared to existing algorithms, the MMT-sJITS does not use a multi-target joint data association process. In the joint measurement situations, the MMT-sJITS considered the joint cluster-measurements being followed by other cluster tracks as modified clutters, thus avoiding the influence of joint measurement events. The proposed method utilized the modified smoothing clutter measurement density to obtain the smoothed components state estimate and forward JITS components state estimate. The impact of backward-running JITS multi-tracks reinforced the forward multi-tracks to obtain the MMT-sJITS state estimation for an effective MMT tracking. This significantly improved FTD performance and accuracy in the estimation compared to other methods.

## Figures and Tables

**Table 1:** Retention parameters and data.

Method	Case	Okay	Swapped	Lost	Result	Execution Time [s]
MMT-sJITS	998	932	64	2	979	1.64
FIsJITS	981	893	75	13	970	1.72
FIsJIPDA	986	931	43	12	972	2.0
JITS	955	897	29	29	946	0.7
JIPDA	956	772	156	28	956	0.5

## Data Availability

The research data presented in this study is available on request from the corresponding author.
